# Supplementary material for: Early events in amyloid-β self-assembly probed by time-resolved solid state NMR and light scattering
Source: Nat Commun. 2023 May 23;14:2964. doi: 10.1038/s41467-023-38494-6 (PMC10205749; doi:10.1038/s41467-023-38494-6)
Supplement: Supplementary file 1 — Supplementary Information [file 41467_2023_38494_MOESM1_ESM.pdf]

SUPPLEMENTARY INFORMATION

Early events in amyloid-β self-assembly probed by time-resolved solid state NMR and light scattering

Jaekyun Jeon, Wai-Ming Yau, and Robert Tycko*
Laboratory of Chemical Physics
National Institute of Diabetes and Digestive and Kidney Diseases
National Institutes of Health
Bethesda, MD 20892-0520
U.S.A.

*corresponding author: Dr. Robert Tycko, National Institutes of Health, Building 5, Room 409, Bethesda, MD 20892-0520, USA. phone: 301-402-8272. email: robertty@mail.nih.gov

Contents:

Supplementary Table 1, page 2

Supplementary Figures 1-9, pages 3-12

Supplementary Table 1: Experimental conditions used to achieve τ_e values from 0.7 ms to 1.0 h.

τ_e (ms)	Total flow rate (ml/min)	Distance from nozzle to copper surface (cm)	Additional volume before nozzle (µl)	Flow hold time
0.7 ms	3.0	0.5	-	-
1.0 ms	3.0	0.5	-	-
1.5 ms	2.0	0.5	-	-
23 ms	1.0	7.2	0.2	-
100 ms	1.0	0.5	1.7	-
200 ms	1.0	0.5	3.3	-
400 ms	1.0	0.5	6.6	-
30 s	1.0	0.5	240	30 s
1.0 h	1.0	0.5	240	1.0 h

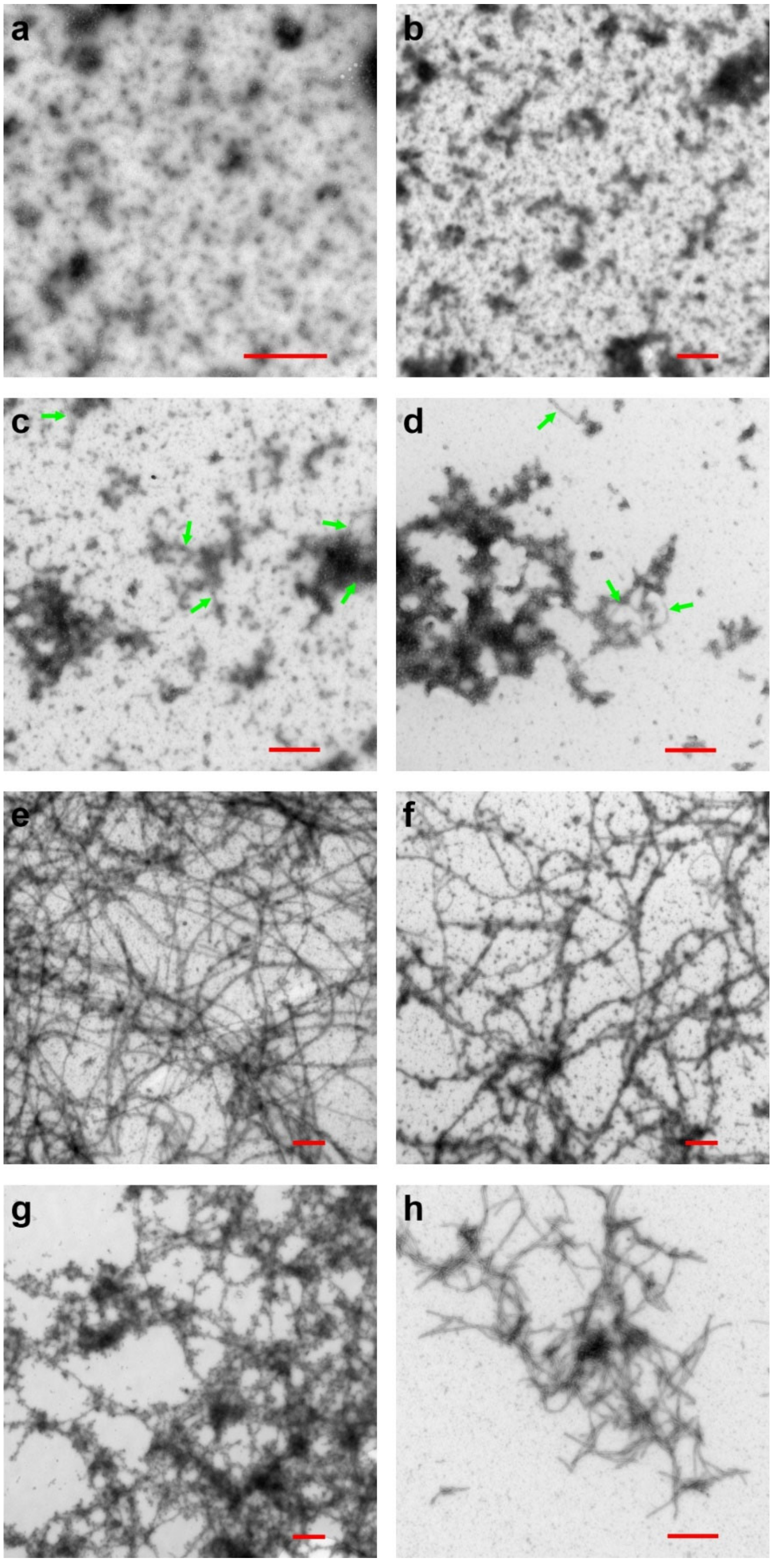

Supplementary Figure 1: Negative-stain TEM images of Aβ40 assemblies. Red scale bars are 500 nm. (a,b) An Aβ40 solution at pH 12 was manually mixed with concentrated sodium phosphate buffer to create a final solution containing 1.5 mM Aβ40 and 175 mM sodium phosphate at pH 7.4. Solutions contained 20% v/v glycerol, as in samples for time-resolved ssNMR. Images were obtained from a TEM grid that was prepared after a 20 min period of quiescent incubation (from a set of 19 micrographs). Assemblies have predominantly nonfibrillar morphologies. (c,d) Images of a TEM grid that was prepared from the same Aβ40 solution after a 1.0 h period of quiescent incubation (from a set of 32 micrographs). Assemblies are still predominantly nonfibrillar, but fibrils are also present (green arrows). (e) Image after a 20 min incubation period, obtained from an Aβ40 solution that was prepared in the same way as in panels a-d, but without glycerol (from a set of 17 micrographs). (f,g) Images from the same Aβ40 solution after a 1.0 h incubation period (from a set of 18 micrographs). Fibrils and nonfibrillar assemblies are present, with apparently similar total masses. (h) Image of fibrils that were prepared without glycerol, after an initial 1.0 h incubation period, brief sonication, and a final 18 h incubation period (from a set of 10 micrographs). These fibrils were used for ssNMR measurements in Figs. 2 and 3 of the main text.

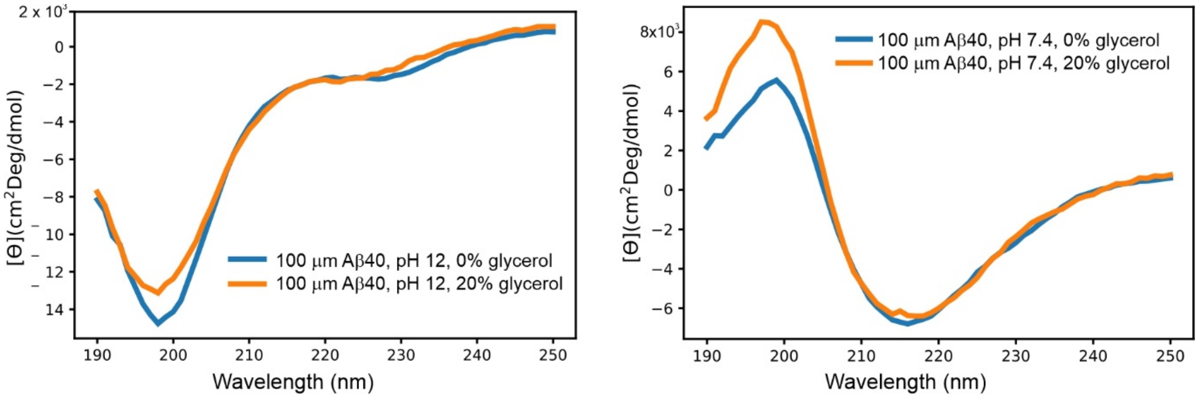

Supplementary Figure 2: Dependence of Aβ40 conformation on pH. Circular dichroism spectra of 100 μm Aβ40 solutions with and without 20% by volume glycerol were recorded at pH 12 (left) and pH 7.4 (right). Spectra at pH 7.4 were acquired at 20° C within 10 min of mixing pH 12 solutions (20 mM NaOH) with concentrated sodium phosphate buffer to achieve a final phosphate concentration of 175 mM. Source data are provided as a Source Data file.

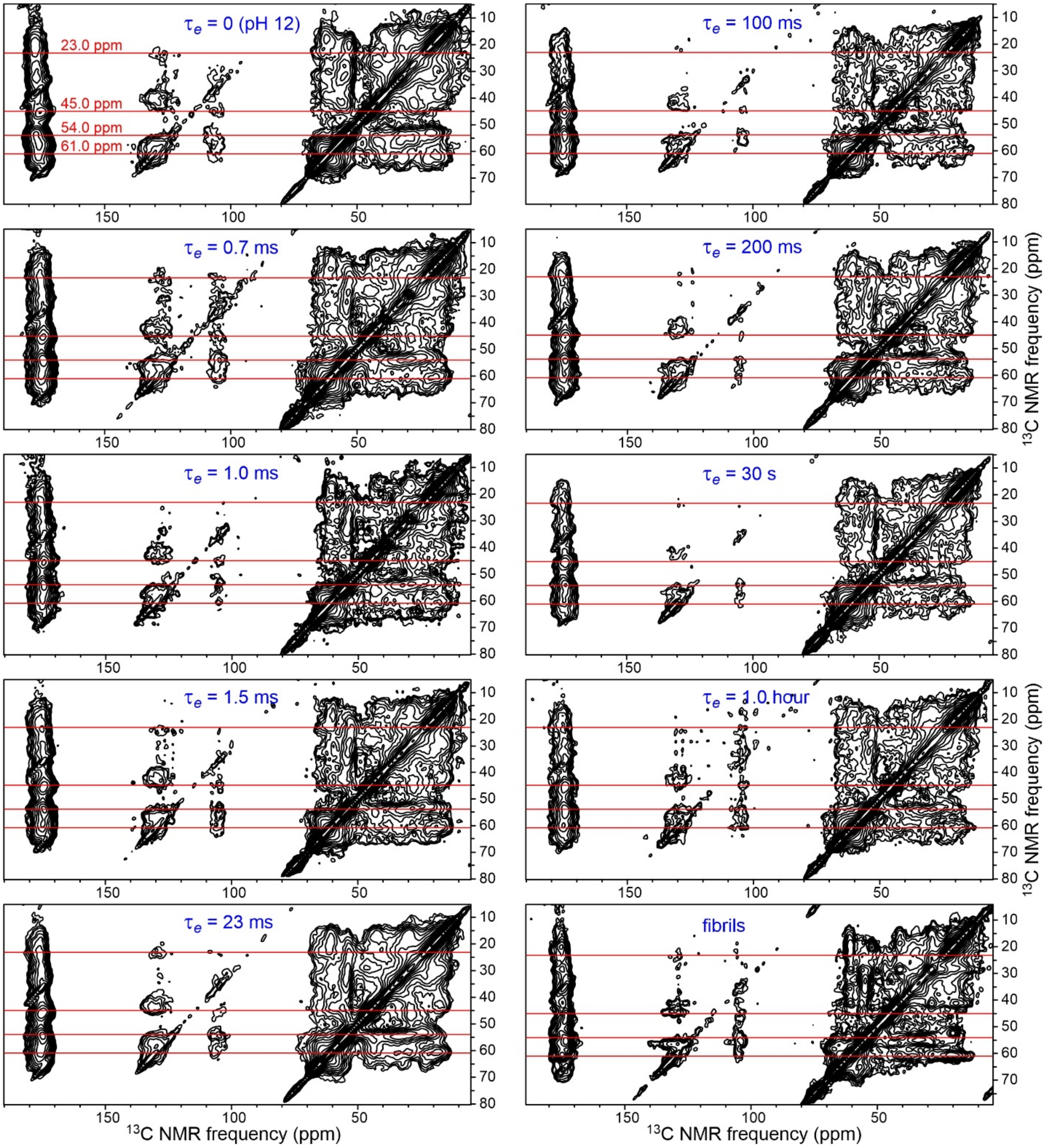

Supplementary Figure 3: Full set of 2D ^{13}C-^{13}C ssNMR spectra of frozen Aβ40-FVGSAILM solutions. 2D spectra with 20 ms mixing periods were obtained for samples with [Aβ40] = 1.5 mM and the indicated values of the evolution time τ_e. Contour levels increase by factors of 1.3. Horizontal red lines indicate positions of 1D slices shown in Supplementary Fig. 4.

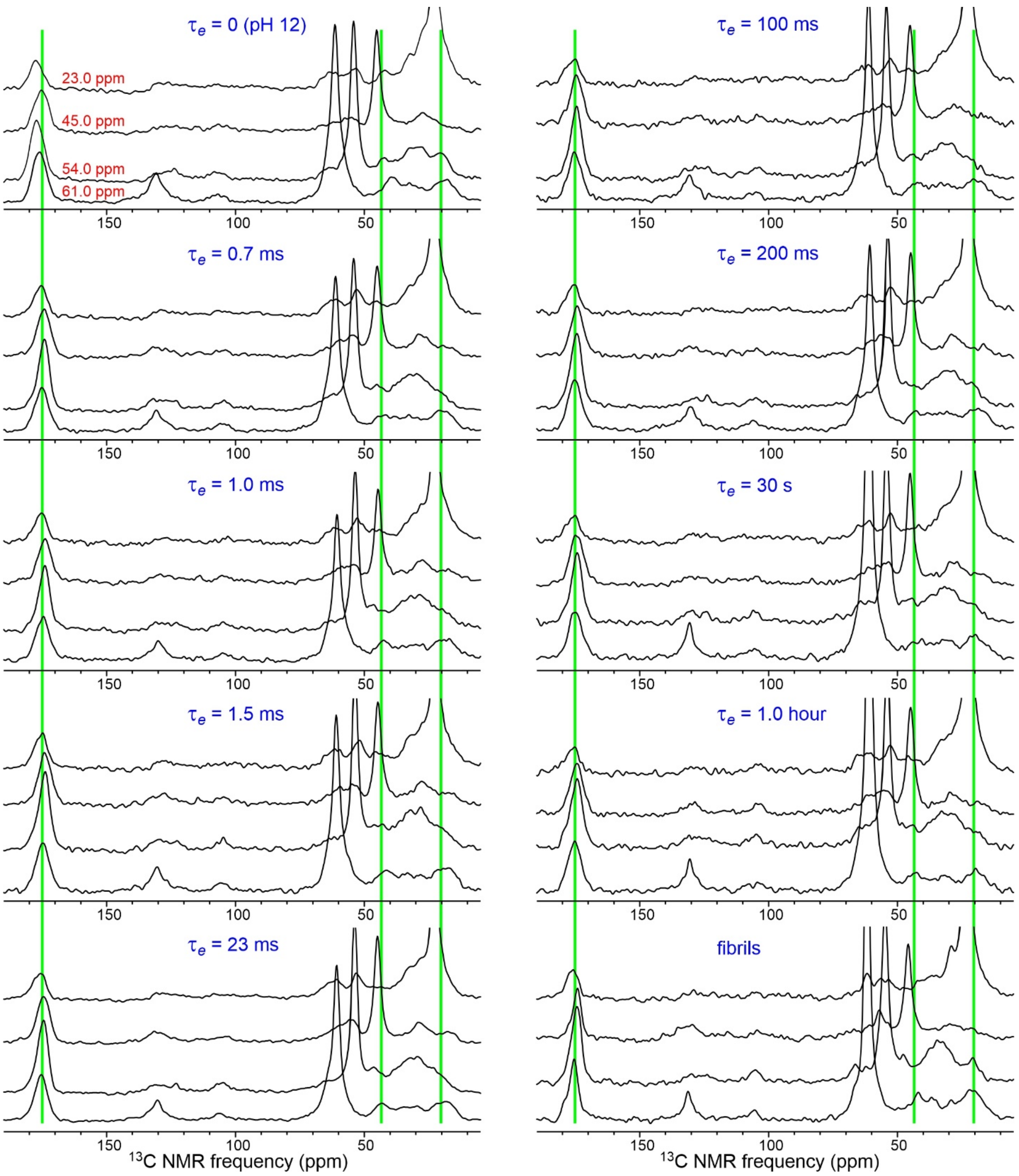

Supplementary Figure 4: 1D slices from time-resolved 2D ssNMR spectra. 1D slices at 23.0 ppm, 45.0 ppm, 54.0 ppm, and 61.0 ppm were extracted from the 2D spectra in Supplementary Fig. 3. Vertical green lines are included to facilitate comparisons of data with different values of the evolution time τ_e.

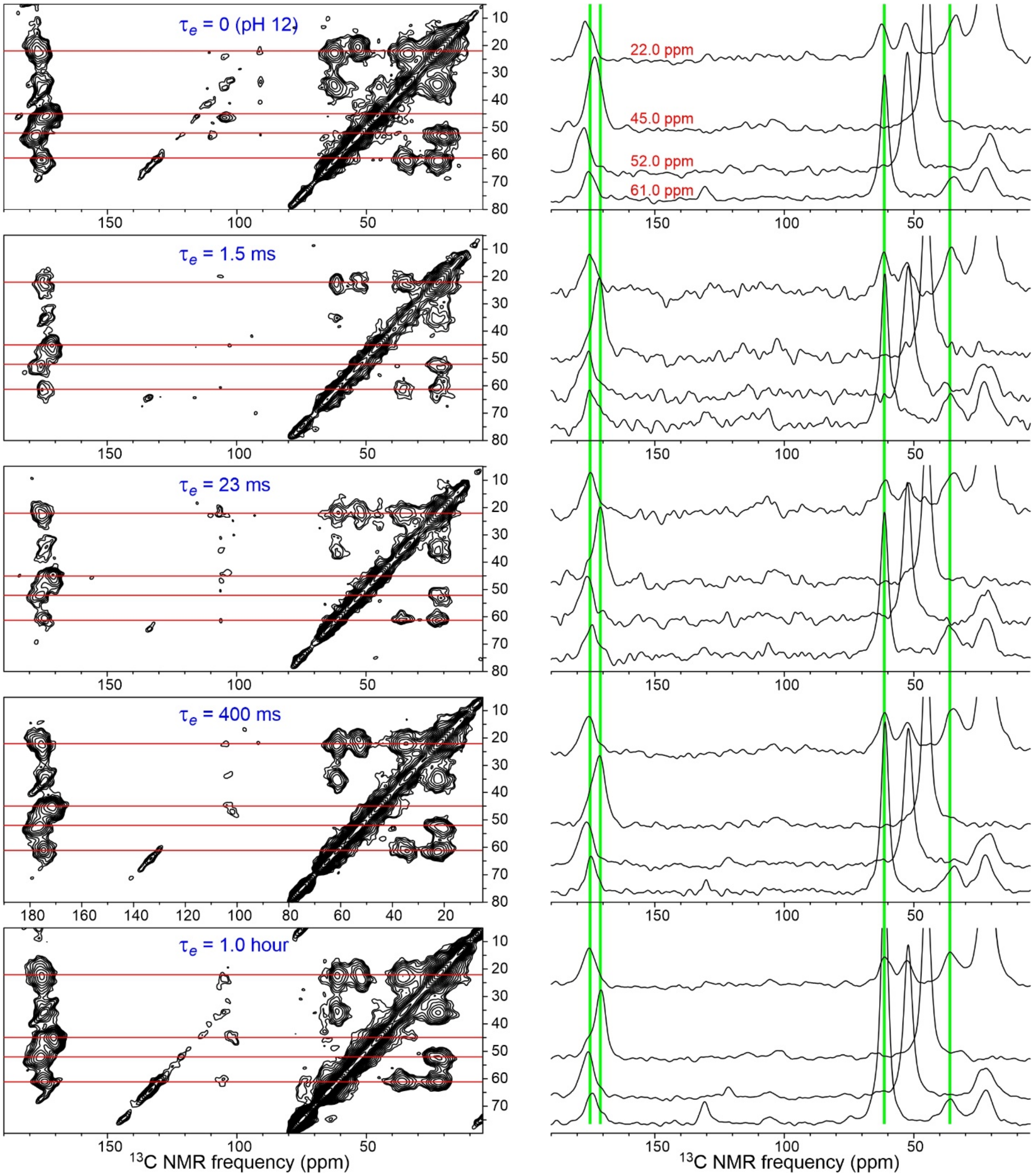

Supplementary Figure 5: Full set of 2D ^{13}C-^{13}C ssNMR spectra of frozen Aβ40-VAG solutions. 2D spectra with 20 ms mixing periods were obtained for samples with [Aβ40] = 1.5 mM and the indicated values of the evolution time τ_e. Contour levels increase by factors of 1.3. 1D slices at the positions indicated by horizontal red lines are shown to the right of each 2D spectrum. Vertical green lines are included to facilitate comparisons of data with different τ_e values.

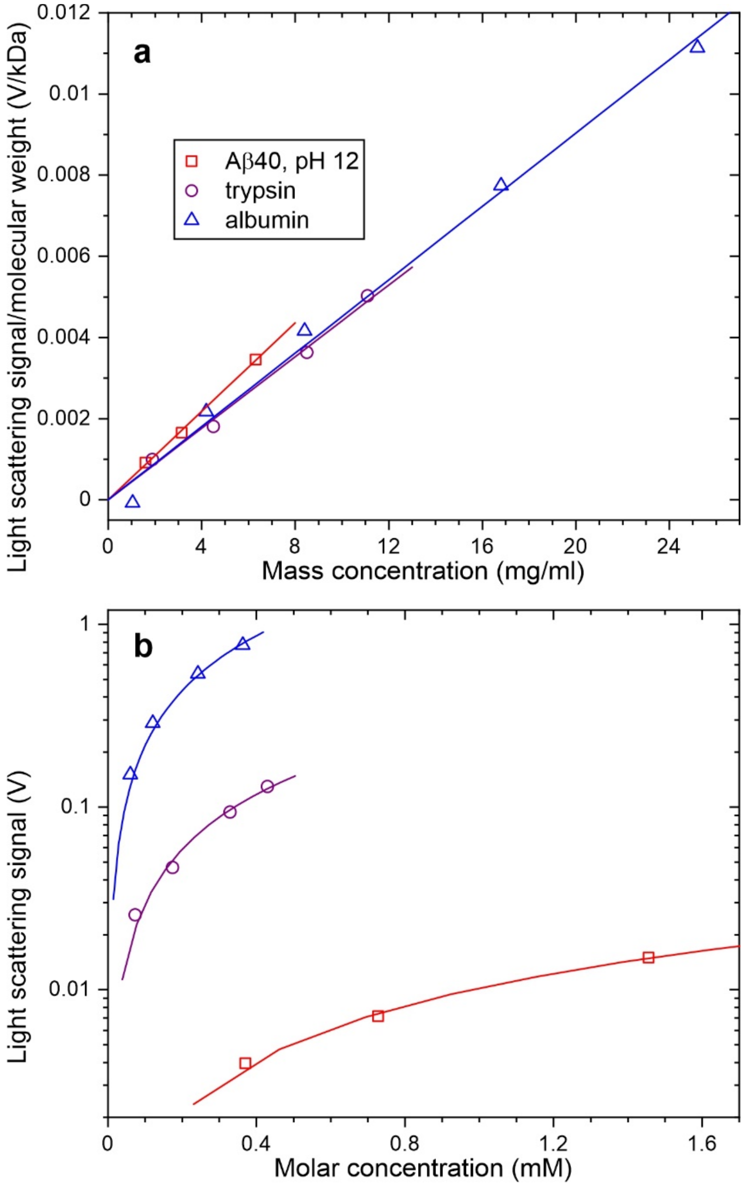

Supplementary Figure 6: Calibrations of light scattering intensities. Light scattering data were obtained for solutions of Aβ40 in 20 mM NaOH, pH 12, bovine trypsin in 50 mM sodium phosphate, pH 7.4, and bovine albumin in 50 mM HEPES buffer, pH 7.4. Data were recorded at 562 nm with the stopped-flow instrument used in time-resolved scattering measurements. Scattering signals were averaged over 0.5 s scans, as in Figs. 5a and 5b of the main text. (a) Data plotted as the scattering signal divided by the protein molecular weight vs. protein mass concentration. Molecular weight (MW) values are 4.33 kDa, 25.8 kDa, and 69.3 kDa for Aβ40, trypsin, and albumin, respectively. Lines are linear fits, yielding slopes of $(5.5 \pm 0.2) \times 10^{-4}$, $(4.4 \pm 0.3) \times 10^{-4}$, and $(4.5 \pm 0.2) \times 10^{-4}$, respectively, with units of V-ml/mg-kDa. In this plot, scattering contributions from solvents were removed by subtracting the y-intercepts of the linear fits. (b) Same data and linear fits, plotted as the light scattering signal vs. protein molar concentration with a logarithmic vertical axis. Source data are provided as a Source Data file.

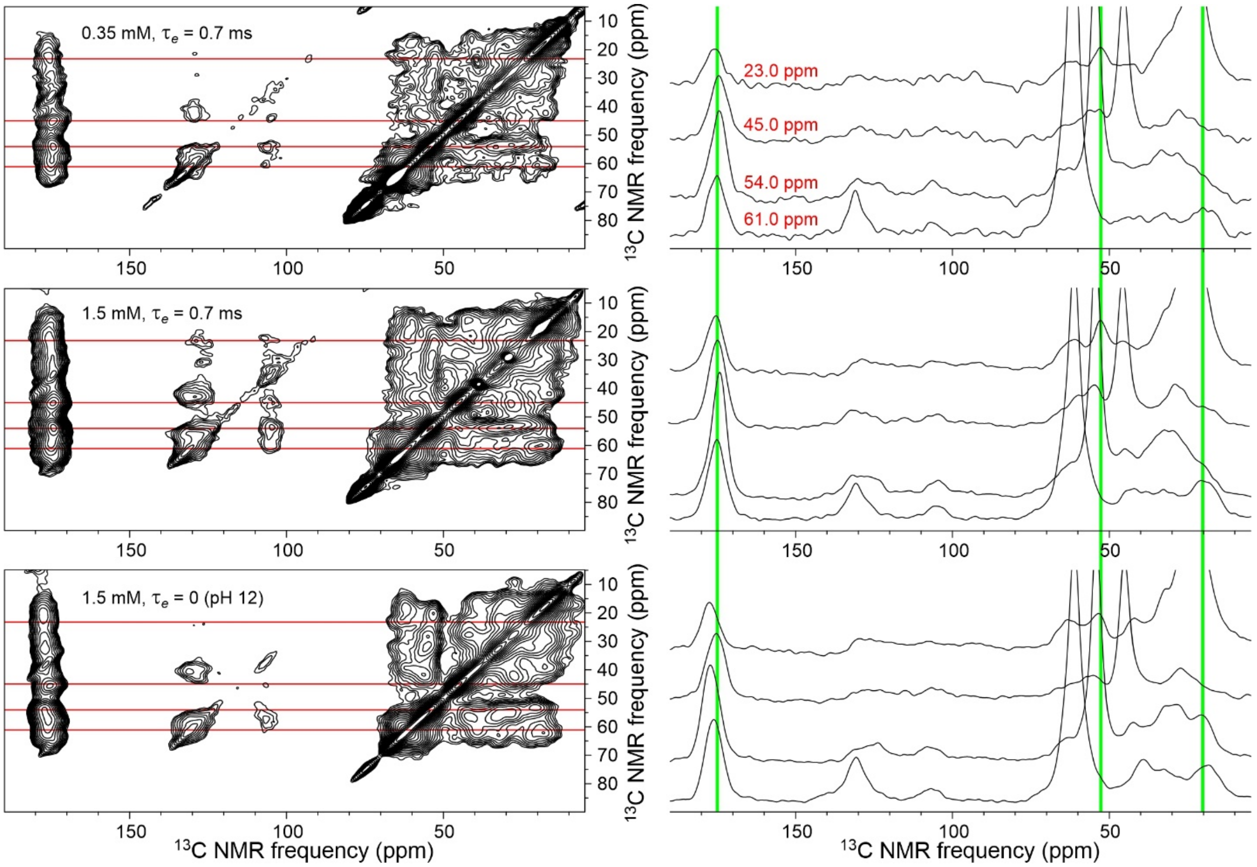

Supplementary Figure 7: Dependence of time-resolved ssNMR data on Aβ40 concentration. The 2D ^{13}C-^{13}C ssNMR spectrum of a frozen Aβ40-FVGSAILM solution with evolution time τ_e = 0.7 ms and [Aβ40] = 0.35 mM (top) is compared with spectra of frozen solutions with [Aβ40] = 1.5 mM and τ_e = 0.7 ms (middle) or τ_e = 0 (bottom). Contour levels increase by factors of 1.2. 1D slices at the positions indicated by horizontal red lines are shown to the right of each 2D spectrum. Vertical green lines are included to facilitate comparisons of data from different samples.

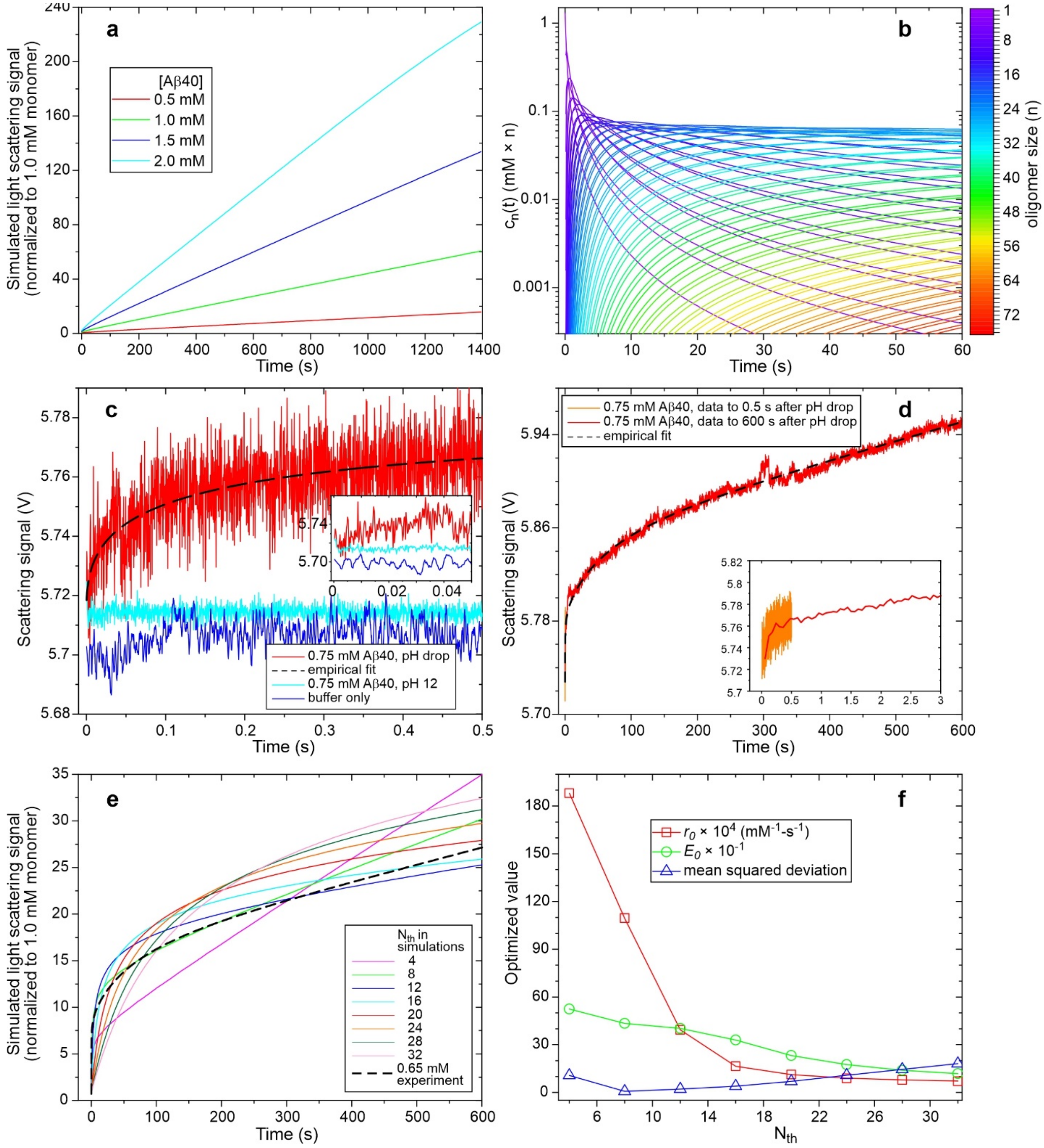

Supplementary Figure 8: Additional light scattering data and simulations. (a) Simulated light scattering data from the coagulation model described in the text with r_0 = 0.01 mM^{-1}s^{-1} and E_0 = 1.0, *i.e.*, with no enhancement of the rates of fusion for small oligomers. Simulated scattering signals are reported in units of the signal from a 1.0 mM solution of Aβ40 monomers. (b) Time dependences of the mass concentrations of Aβ40 *n*-mers with specific values of *n*, from simulations with oligomer fusion rate r_0 = 0.0054 mM^{-1}s^{-1}, enhancement factor E_0 = 112.6, size threshold N_{th} = 16, and an initial monomer concentration of 1.5 mM. These parameter values represent the best fit to the experimental data in Fig. 5b. Oligomer mass concentrations $c_n(t)$ are

divided by the molecular weight of the monomer, resulting in units of mM×n. Results are shown for $1 \leq n \leq 76$. (c) Experimental light scattering signals for a 0.75 mM Aβ40 solution at pH 12 (cyan), a 0.75 mM Aβ40 solution after a rapid pH drop from 12 to 7.4 (red), and a pH 7.4 buffer alone (blue). Dashed line is a fit with the expression $S(t) - S_b = A_1 + B_1\{1 - \exp[-(t/\tau_1)^{\beta_1}]\}$, yielding A_1 = 0.0066 V, B_1 = 0.0643 ± 0.0053 V, τ_1 = 152 ± 55 ms, and β_1 = 0.422 ± 0.037. Inset shows the data up to 50 ms. (d) Light scattering data recorded to 600 s after a rapid pH drop. Inset compares the data up to 3.0 s (red) with pH drop data from panel c (orange). Dashed line is a fit with the expression $S(t) - S_b = A_1 + A_2 t + B_1\{1 - \exp[-(t/\tau_1)^{\beta_1}] + B_2\{1 - \exp[-(t/\tau_2)^{\beta_2}]\}$, using values of A_1, B_1, τ_1, and β_1 determined in panel c. Best-fit values of other parameters are A_2 = 0.0001623 ± 0.0000012 V/s, B_2 = 0.07527 ± 0.00068 V, τ_2 = 57.3 ± 1.2 s, and β_2 = 0.7342 ± 0.0084. (e) Fits of the experimental data, represented by the dashed line, with simulations based on the coagulation model described in the text. Simulation parameters r_0 and E_0 were optimized for each value of N_{th}. (d) Dependences of the optimized values of r_0 and E_0 and the deviation between optimized simulations and experimental data on N_{th}. The best agreement between experimental and simulated data is observed with $N_{th} \approx$ 8-12, $r_0 \approx$ 0.011-0.0040, and $E_0 \approx$ 420. Source data are provided as a Source Data file.

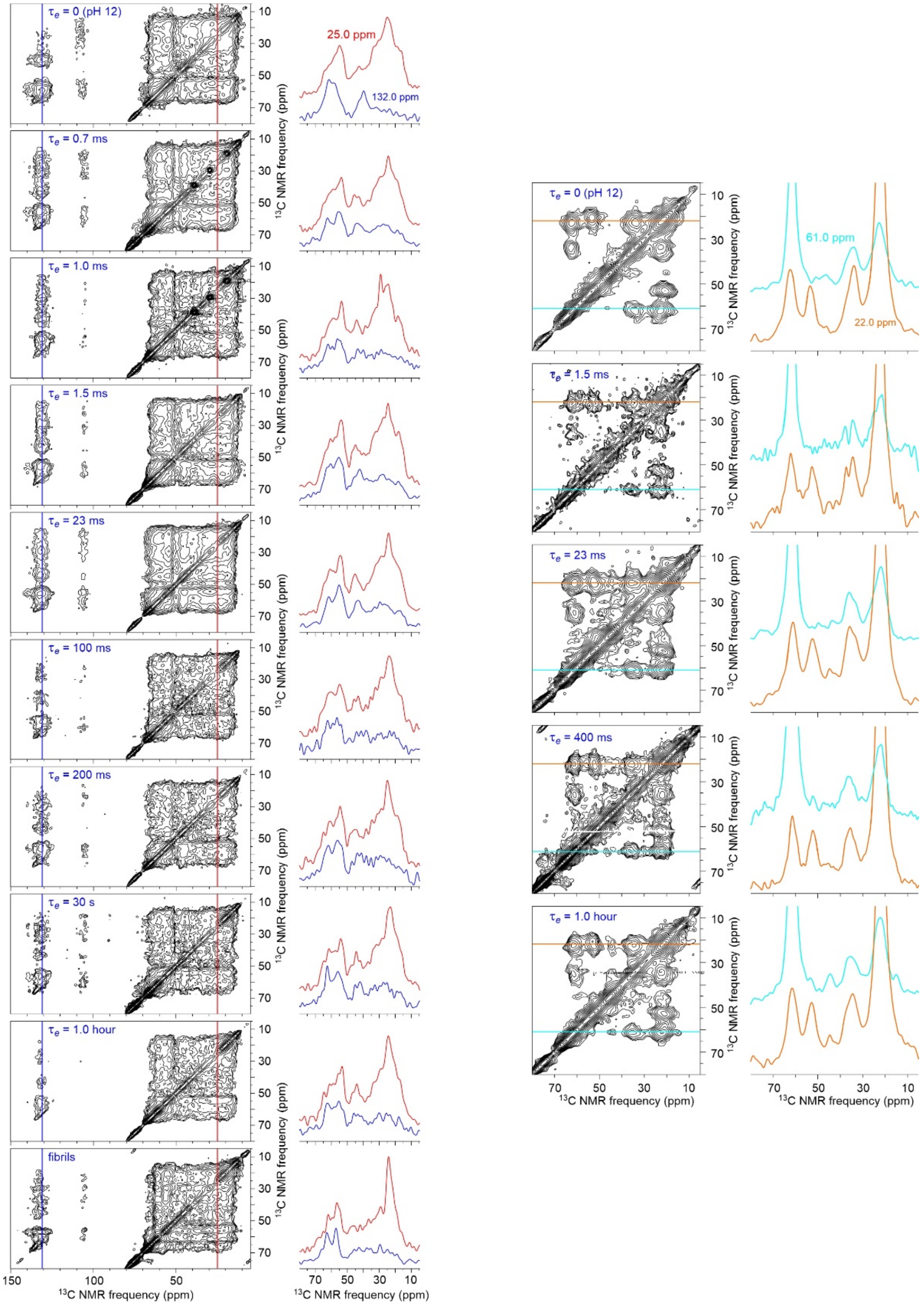

Supplementary Figure 9: Full sets of 2D ^{13}C-^{13}C ssNMR spectra with 1.0 s mixing periods. 2D spectra of frozen Aβ40-FVGSAILM (a) and Aβ40-VAG (b) solutions are shown for the indicated values of the evolution time τ_e. 1D slices at the positions indicated by vertical red and blue or horizontal cyan and orange lines are shown to the right of each 2D spectrum. Contour levels increase by factors of 1.2.
