# Supplementary material for: Early events in amyloid-β self-assembly probed by time-resolved solid state NMR and light scattering
Source: Nat Commun. 2023 May 23;14:2964. doi: 10.1038/s41467-023-38494-6 (PMC10205749; doi:10.1038/s41467-023-38494-6)
Supplement: Supplementary file 3 — Reporting Summary [file 41467_2023_38494_MOESM3_ESM.pdf]

nature portfolio

Corresponding author(s): Robert Tycko

Last updated by author(s): 04/21/2023

Reporting Summary

Nature Portfolio wishes to improve the reproducibility of the work that we publish. This form provides structure for consistency and transparency in reporting. For further information on Nature Portfolio policies, see our Editorial Policies and the Editorial Policy Checklist.

Statistics

For all statistical analyses, confirm that the following items are present in the figure legend, table legend, main text, or Methods section.

n/a	Confirmed	
☐	☒	The exact sample size (*n*) for each experimental group/condition, given as a discrete number and unit of measurement
☒	☐	A statement on whether measurements were taken from distinct samples or whether the same sample was measured repeatedly
☒	☐	The statistical test(s) used AND whether they are one- or two-sided<br>*Only common tests should be described solely by name; describe more complex techniques in the Methods section.*
☒	☐	A description of all covariates tested
☒	☐	A description of any assumptions or corrections, such as tests of normality and adjustment for multiple comparisons
☐	☒	A full description of the statistical parameters including central tendency (e.g. means) or other basic estimates (e.g. regression coefficient) AND variation (e.g. standard deviation) or associated estimates of uncertainty (e.g. confidence intervals)
☒	☐	For null hypothesis testing, the test statistic (e.g. *F*, *t*, *r*) with confidence intervals, effect sizes, degrees of freedom and *P* value noted<br>*Give P values as exact values whenever suitable.*
☒	☐	For Bayesian analysis, information on the choice of priors and Markov chain Monte Carlo settings
☒	☐	For hierarchical and complex designs, identification of the appropriate level for tests and full reporting of outcomes
☒	☐	Estimates of effect sizes (e.g. Cohen's *d*, Pearson's *r*), indicating how they were calculated

Our web collection on statistics for biologists contains articles on many of the points above.

Software and code

Policy information about availability of computer code

Data collection	NMR data were acquired with Bruker Topspin 3.2 software. Light scattering and fluorescence data were acquired with the Pro-Data SX software (version 2.5.1852.0)of the Applied Photophysics stopped flow instrument. CD data were acquired with the Spectra Manager software (version 2.13.00) of the Jasco spectrometer.
Data analysis	NMR data were processed with nmrPipe software (version 9.4, Rev. 2017.335.16.23), plotted with nmrDraw software (also version 9.4, Rev. 2017.335.16.23) and Sparky software (version 3.114), and analyzed with Python scripts. Fits of light scattering data were performed in Origin 2020b software (version 9.7.5.184). Simulations and fitting of light scattering data based on the coagulation model described in this paper were performed with custom Fortran95 programs, which have been deposited at https://doi.org/10.17632/hkzth2dmms.1.

For manuscripts utilizing custom algorithms or software that are central to the research but not yet described in published literature, software must be made available to editors and reviewers. We strongly encourage code deposition in a community repository (e.g. GitHub). See the Nature Portfolio guidelines for submitting code & software for further information.

Data

Policy information about availability of data

All manuscripts must include a data availability statement. This statement should provide the following information, where applicable:

- Accession codes, unique identifiers, or web links for publicly available datasets
- A description of any restrictions on data availability
- For clinical datasets or third party data, please ensure that the statement adheres to our policy

2D ssNMR spectra and TEM images are available from https://doi.org/10.17632/kcjbbz9gzs.1. Raw data from light scattering, circular dichroism, and ThT fluorescence measurements are now included in the Source Data file. These are the complete data sets from measurements described in this manuscript. However, in the event that readers want additional information, other data are available from the authors upon request.

Human research participants

Policy information about studies involving human research participants and Sex and Gender in Research.

Reporting on sex and gender	N/A
Population characteristics	N/A
Recruitment	N/A
Ethics oversight	N/A

Note that full information on the approval of the study protocol must also be provided in the manuscript.

Field-specific reporting

Please select the one below that is the best fit for your research. If you are not sure, read the appropriate sections before making your selection.

☒ Life sciences ☐ Behavioural & social sciences ☐ Ecological, evolutionary & environmental sciences

For a reference copy of the document with all sections, see nature.com/documents/nr-reporting-summary-flat.pdf

Life sciences study design

All studies must disclose on these points even when the disclosure is negative.

Sample size	No sample size calculations were performed, as such calculations are not relevant to the experiments described in this manuscript. A single sample contains approximately 10,000,000,000,000,000 amyloid-beta molecules. Sample size considerations of the type that pertain to life sciences studies, such as experiments on animal models (e.g., mice), are irrelevant to the statistical validity of our results. Instead, the statistical validity depends on the signal-to-noise ratio of the data, which is determined by the quantity of material within each sample and the measurement times. Sample quantities (i.e., volumes of material) and concentrations of isotopically labeled peptides were chosen to give adequate signal-to-noise ratios in ssNMR measurements within measurement periods of 6 hours or less for each sample (i.e., for each evolution time point in the time-resolved ssNMR experiments).
Data exclusions	No data were excluded.
Replication	In the time-resolved ssNMR measurements, multiple samples were prepared independently with different structural evolution periods and labeling patterns. A total of 16 frozen solutions with different evolution periods and labeling patterns were prepared independently (on different days) and used for 2D ssNMR measurements with two different conditions (20 ms and 1000 ms 13C-13C mixing periods in the 2D pulse sequence), for a total of 32 2D ssNMR spectra. No attempt was made to make multiple samples with precisely the same evolution times and labeling patterns. However, results were found to be consistent, as shown in the manuscript, in that the dependences of ssNMR signals on the structural evolution period showed no evidence for random or inexplicable variations. In stopped flow light scattering and fluorescence measurements, scans were repeated until the system was thoroughly flushed and a constant baseline was established, after which signals in six successive scans were found to be consistent and were averaged together.
Randomization	Allocation to groups or randomization was not relevant to the experiments described in this manuscript, as these experiments did not seek to distinguish between "control" and "treatment" groups.
Blinding	Blinding was not performed, since our experiments were not of a type where blinding is a meaningful or practical concept. Samples were prepared and measurements on these samples were performed by a single individual, as is often the case in the fields of biophysics, biophysical chemistry, and structural biology.

Reporting for specific materials, systems and methods

We require information from authors about some types of materials, experimental systems and methods used in many studies. Here, indicate whether each material, system or method listed is relevant to your study. If you are not sure if a list item applies to your research, read the appropriate section before selecting a response.

Materials & experimental systems

n/a	Involved in the study
☒	☐ Antibodies
☒	☐ Eukaryotic cell lines
☒	☐ Palaeontology and archaeology
☒	☐ Animals and other organisms
☒	☐ Clinical data
☒	☐ Dual use research of concern

Methods

n/a	Involved in the study
☒	☐ ChIP-seq
☒	☐ Flow cytometry
☒	☐ MRI-based neuroimaging
